# Evolution of trees and mycorrhizal fungi intensifies silicate mineral weathering

**DOI:** 10.1098/rsbl.2012.0503

**Published:** 2012-08-01

**Authors:** Joe Quirk, David J. Beerling, Steve A. Banwart, Gabriella Kakonyi, Maria E. Romero-Gonzalez, Jonathan R. Leake

**Affiliations:** 1 Department of Animal and Plant Sciences, University of Sheffield, Sheffield, S10 2TN, UK; 2 Kroto Research Institute, University of Sheffield, North Campus, Sheffield S3 7HQ, UK

**Keywords:** biological weathering, arbuscular mycorrhiza, ectomycorrhiza, land plant evolution, silicate mineral weathering, global change ecology

## Abstract

Forested ecosystems diversified more than 350 Ma to become major engines of continental silicate weathering, regulating the Earth's atmospheric carbon dioxide concentration by driving calcium export into ocean carbonates. Our field experiments with mature trees demonstrate intensification of this weathering engine as tree lineages diversified in concert with their symbiotic mycorrhizal fungi. Preferential hyphal colonization of the calcium silicate-bearing rock, basalt, progressively increased with advancement from arbuscular mycorrhizal (AM) to later, independently evolved ectomycorrhizal (EM) fungi, and from gymnosperm to angiosperm hosts with both fungal groups. This led to ‘trenching’ of silicate mineral surfaces by AM and EM fungi, with EM gymnosperms and angiosperms releasing calcium from basalt at twice the rate of AM gymnosperms. Our findings indicate mycorrhiza-driven weathering may have originated hundreds of millions of years earlier than previously recognized and subsequently intensified with the evolution of trees and mycorrhizas to affect the Earth's long-term CO_2_ and climate history.

## 1. Introduction

Forested ecosystems are major engines of biological weathering in terrestrial environments, but we know almost nothing about how the strength of these engines changed as tree lineages and their root-associating fungal symbionts evolved. Fossil roots of early gymnosperms from at least the Carboniferous are colonized by arbuscular mycorrhizal (AM) fungi, and this type of mycorrhiza continues to be found in the vast majority of tree species, including in most of the more recently evolved angiosperm taxa [1]. Independently evolving ectomycorrhizal (EM) fungi diversified from the Cretaceous, forming mycorrhizal associations with the Pinaceae and angiosperm trees in the Betulaceae and Fagaceae that now dominate temperate and boreal forests, as well as with angiosperm trees in the Myrtaceae, Fabaceae and Dipterocarpaceae, that can form dominant stands in warm temperate and tropical regions [1,2]. Both mycorrhizal types use host photosynthate to support extensive hyphal networks with high absorptive surface area for nutrient element mass transfer from the substrate. In trees forming AM, root functioning is augmented by the nutrient-scavenging activities of the fungi, whereas EM fungi completely envelop tree root tips to subsume the soil–root interface. EM fungi thereby control the translocation of elements from soil to tree and can also enhance mineral weathering through exudation of low molecular weight organic compounds [3,4].

**Figure 1.**
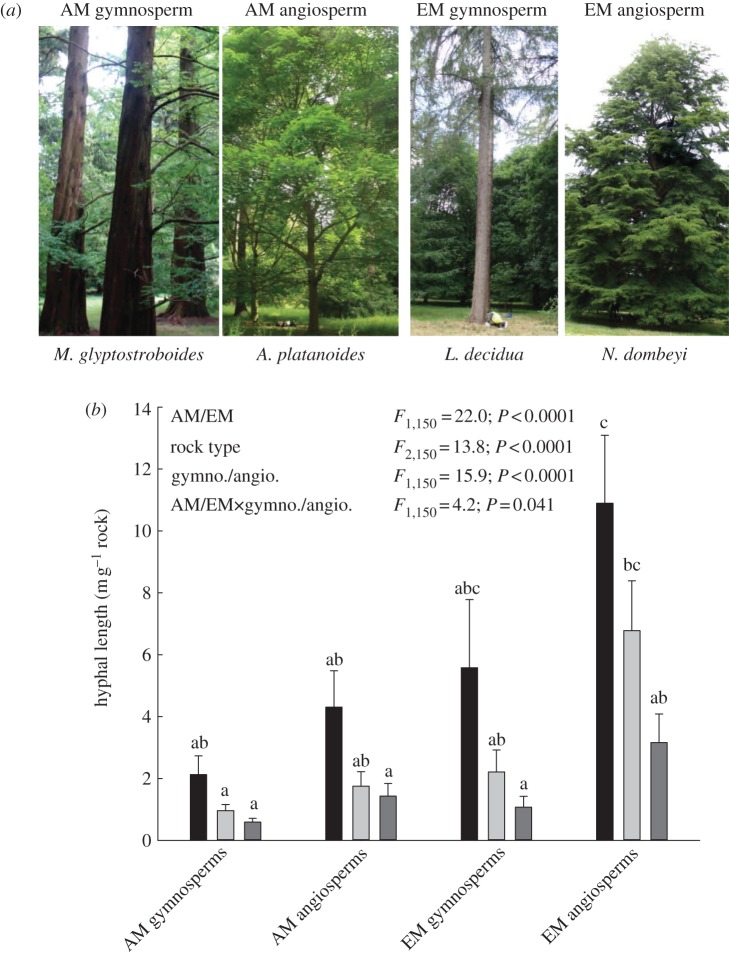
Fungal colonization of rock grains. (*a*) Representative trees in established stands at Westonbirt Arboretum (photographs J.Q). (*b*) Mean ± s.e.m. hyphal lengths colonizing rock grains after five months (*n* = (7 + 7) = 14 trees from two species; table 1). Bars (black, basalt; light grey, granite; dark grey, quartz) sharing the same letter are not statistically different (*p* < 0.05). Tree leaf habit (deciduous/evergreen) had no effect on colonization (see electronic supplementary material, table S3), justifying the pooling of species into major tree–mycorrhiza functional types.

Here, we address the primary hypothesis that functional differences between mycorrhizal types, coupled with the evolution of their host trees, drives intensification of silicate weathering. We used mature tree taxa with crown diversification ages ranging from tens to hundreds of millions of years (figure 1*a* and table 1) in conjunction with a suite of methods isolating mycorrhizal hyphal effects on mineral weathering by excluding tree roots with mesh bags [8]. The extant gymnosperm taxa available for these studies may be only approximate representatives of the ancestral taxa that dominated temperate forests before the rise to dominance of angiosperms [6,9]. Stem- and crown-node ages estimated with molecular clocks suggest gymnosperms evolved and adapted over the same evolutionary time span as their sister lineages, the angiosperms (table 1) [6]*.* Mycorrhiza-driven weathering was quantified by burying uniform-sized grains of silicate rocks that are either calcium-rich (basalt) or -poor (granite), along with quartz controls (see the electronic supplementary material, tables S1 and S2). Weathering of calcium from silicates plays a major role in regulating atmospheric CO_2_ on geological timescales [10,11] by promoting the deposition of marine calcium carbonates. Our field studies control for climate and soil type by focusing on established trees with natural populations of soil micro-organisms at the National Arboretum, Westonbirt, UK.

**Table 1.** Tree species used to study mycorrhiza-driven weathering. Tree group: gymnosperm (G) or angiosperm (A); leaf habit: evergreen (E) or deciduous (D).

mycorrhiza	tree group	leaf habit	species	family	stem–crown node age (Ma)^c^	mean height (m)	mean DBH (m)^d^
AM (more than 400 Ma^a^)	G	E	*Sequoia sempervirens* D. Don.	Cupressaceae	190–163	34 ± 1.6	1.1 ± 0.11
	G	D	*Metasequoia glyptostroboides* Hu & Cheng	Cupressaceae	190–163	26 ± 2.5	0.6 ± 0.09
	A	E	*Ilex aquifolium* L.^e^	Aquifoliaceae	65–52	8 ± 1.3	0.2 ± 0.02
	A	D	*Acer platanoides* L.^e^	Sapindaceae	55–36	22 ± 0.6	0.4 ± 0.01
EM (220–135 Ma^b^)	G	E	*Pinus sylvestris* L.	Pinaceae	263–100	32 ± 2.1	0.7 ± 0.05
	G	D	*Larix decidua* Mill.^e^	Pinaceae	263–100	34 ± 1.5	0.6 ± 0.03
	A	E	*Nothofagus dombeyi* Mirb.	Nothofagaceae	61–36	21 ± 1.7	0.5 ± 0.07
	A	D	*Betula pendula* Roth.	Betulaceae	36–25	20 ± 0.7	0.4 ± 0.01

^a^See [5].

^b^From [2,4].

^c^Stem and crown node ages as given: Cupressaceae and Pinaceae [6]; Sapindaceae, Aquifoliaceae, Betulaceae and Fagaceae (*Nothofagus*) using maximum likelihood to calculate branch length [7].

^d^Mean ± s.e.m. trunk diameter at breast height (DBH; *n* = 7).

^e^Individuals felled after five months as part of Arboretum management.

## 2. Methods

Replicate trees (*n* = 7) for each of our eight species were identified at Westonbirt Arboretum (figure 1*a* and table 1; electronic supplementary material, methods: field experiment). Hyphal in-growth mesh bags (35 µm pore-size) containing 2.5 g of crushed (0.25–1.00 mm grain-size) Tertiary basalt (Northern Ireland), Shap granite (Cumbria, UK) or high-purity quartz (see the electronic supplementary material, methods: test rocks) was buried 1 m from the base of each trunk in June 2009 at 10 cm depth in the A-horizon (see electronic supplementary material, methods: rock-filled mesh bags). Sets of bags (*n* = 7 per rock per species) were recovered after five months and hyphal colonization of the grains was determined using standard techniques [8] (see electronic supplementary material, methods: hyphal lengths colonizing rocks). Further sets of basalt bags (*n* = 7 per rock per species) for five species (table 1) were recovered after 14 months, their pH measured and the contents subjected to sequential chemical extraction of the exchangeable (1 M ammonium acetate), carbonate (1 M sodium acetate and acetic acid, pH 5.0) and oxide fractions (0.5 M hydroxylamine-hydrochloride in 25% acetic acid followed by 0.1 M ammonium oxalate adjusted to pH 3.0 with 0.2 M oxalic acid and 0.1 M ascorbic acid). Extraction solutions were diluted, acidified with 1 per cent nitric acid and calcium and strontium concentrations determined using inductively coupled plasma mass spectrometry (PerkinElmer Elan DRC II, MA, USA; electronic supplementary material, methods: sequential chemical extractions).

Silicate mineral surface alteration was assessed with muscovite flakes embedded in silicone mounted on 26 × 4 mm glass slides which were buried in mesh bags with 0.5 g of crushed basalt between November 2009 and August 2010. On recovery, mineral surfaces were characterized using vertical scanning interferometry (VSI; Wyko, NT 9100; Bruker, WI, USA; electronic supplementary material, methods: characterization of mineral surfaces). Measurement of the width and depth of surface trenches was undertaken using Vision v. 4.10 software (Bruker, WI, USA). Cross-sectional trench dimensions were obtained from four scans from two muscovite flakes per species where localized fungal-driven mineral degradation was observed (beneath *Sequoia sempervirens*, *Pinus sylvestris* and *Betula pendula*, but not *Metasequoia glyptostroboides* or *Nothofagus dombeyi*).

Statistical testing was conducted in Minitab v. 12.21 following the assumptions of a general linear model factorial design. Hyphal length data were analysed with three-way ANOVA and Tukey multiple comparisons testing for effects of rock type, mycorrhiza and tree functional type (gymnosperm or angiosperm). Mineral trench widths and depths, and calcium dissolution were analysed with one-way ANOVAs and Tukey multiple comparisons testing for effects of species (see electronic supplementary material, methods: statistics).

## 3. Results and discussion

After five months, fungal hyphae associated with AM and EM tree roots preferentially colonized basalt over granite and quartz (figure 1*b*; electronic supplementary material, figure S1 shows comparisons between species), which is indicative of positive feedbacks between elemental uptake from basalt by hyphae in return for increasing carbon provision from tree roots. Furthermore, these hyphal interactions with silicate rock grains intensified with the evolution of tree–mycorrhiza partnerships (figure 1*b*). Hyphae from EM angiosperms demonstrated five-times greater colonization of basalt (Tukey *T*-value = 5.54; *p* < 0.0001) and seven-times greater colonization of granite (Tukey *T*-value = 3.68; *p* = 0.017) than AM gymnosperms (figure 1*b*).

**Figure 2.**
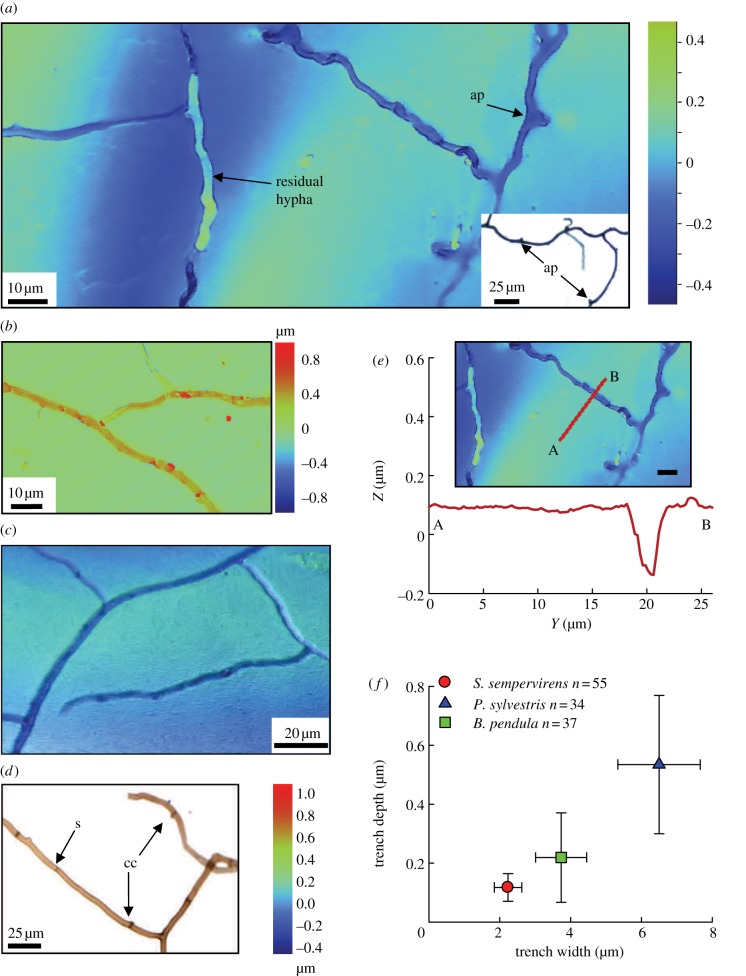
Fungal–mineral interactions. (*a*) Branched trenches on muscovite beneath AM *S. sempervirens* show angular projections (ap) and residual hyphal material; inset shows Trypan blue-stained AM hyphae recovered from basalt beneath *S. sempervirens*. (*b*) Hyphae on muscovite beneath EM *B. pendula* share comparable morphologies with, (*c*), trenches on muscovite beneath the same trees. (*d*) Fungus recovered from basalt beneath *B. pendula*. (*e*) VSI surface profile along a mica transect showing a trench cross-section. (*f*) Mean ± s.d. width and depth of trench cross-sections relative to the surrounding planar surface (*n* = 4 scans from *n* = 2 pieces of muscovite per species) buried beneath *S. sempervirens* (red circle, AM), *P. sylvestris* (blue triangle, EM) and *B. pendula* (green squares, EM). (*e*) Scale bar, 10 µm.

The capacity of this fungal proliferation to physically disrupt silicate mineral surfaces was determined by burying muscovite flakes inside the mesh bags containing basalt. Muscovite is a potassium-bearing aluminium-phyllosilicate allowing microscale topographic surface characterization using VSI. Importantly, in three of the five tree–mycorrhiza partnerships studied (table 1), VSI revealed direct evidence of localized physical alteration and loss of the muscovite where colonizing hyphae contacted the mineral surface (figure 2). Muscovite beneath the AM gymnosperm *S. sempervirens* exhibited branched linear trenches with comparable morphology and dimensions to AM Glomeromycotean fungi recovered from basalt grains beneath the same tree (figure 2*a*). Raised linear structures consistent with branched hyphae on the mineral surface (figure 2*b*), and trenches of a similar form in the muscovite buried beneath the EM trees *B. pendula* and *P. sylvestris* (figure 2*c*), shared similar diagnostic morphology to EM-forming Basidiomycotean fungi colonizing the rock grains beneath the same trees [1] (figure 2*d*). Hyphae from beneath AM trees showed typical Glomeromycotean (AM) fungal characteristics [1], being highly branched with angular projections, generally lacking septa, unpigmented and readily stained with Trypan-blue fungal dye (figure 2*a*). Conversely, hyphae from beneath EM trees were typical of Basidiomycotean fungi, exhibiting septa, clamp connections and melanization [1] (figure 2*d*). Quantitative metrology of muscovite surface trench features revealed that the degree of mineral disruption was dependent on the type of tree–mycorrhiza partnership. Trenches beneath EM trees were two to three-times wider (*F*_2,177_ = 382; *p* < 0.0001) and two to four-times deeper (*F*_2,177_ = 129; *p* < 0.0001) than those on muscovite beneath AM *S. sempervirens* (figure 2*e*,*f*). Cross-plots indicate that the degree of physical alteration varied with mycorrhizal type (figure 2*f*). These field results for EM *Betula* and *Pinus* in natural soils are consistent with *in vitro* observations of *Pinus* seedling EM-driven weathering of a phyllosilicate through biomechanical forcing and chemical dissolution [12].

Sequential chemical extraction of different physico-chemical fractions of the basalt grains provides evidence for the ability of the five major tree–mycorrhiza partnerships (table 1) to mobilize calcium bound within dominant silicates and trace carbonates relative to unweathered controls. The primary calcium sources in basalt are the dominant silicate minerals such as plagioclase. Given minor oxide minerals in the basalt are likely to be iron-bearing oxides, like magnetite that lack calcium, calcium extracted following removal of trace carbonates represents mobilization of silicate-bound calcium into the oxide extraction phase. Calcium dissolution from the oxide and carbonate fractions significantly increased (*p* < 0.0001) beneath EM-angiosperm compared with AM-gymnosperm trees (table 2), consistent with more extensive basalt colonization and surface alteration of silicates by EM hyphae (figure 2*f*). Ratios of Ca : Sr (µmol : µmol) aid diagnosis of the Ca source from the different mineral components of basalt, with low ratios in plagioclase (less than 200) [13], the dominant calcium-bearing silicate mineral phase in the basalt, and high ratios in carbonates (more than 500) [14]. The chemical extractions demonstrate that Ca : Sr from the oxide fraction is an order-of-magnitude lower than from carbonates (table 2), indicating calcium extracted from the oxide fraction during hyphal–basalt interactions was predominantly mobilized from plagioclase.

**Table 2.** Estimated Ca dissolution rates from basalt. *n* = 5–7 ± s.e.m. for trees and *n* = 3 ± s.e.m. for unweathered basalt.

mycorrhiza	tree group	species/treatment	basalt bag solution pH^d^	oxide bound Ca dissolution (ng g^−1^ h^−1^)	carbonate-bound Ca dissolution (ng g^−1^ h^−1^)	Ca : Sr (oxide fraction) (µmol : µmol)	Ca : Sr (carbonate fraction) (µmol : µmol)
none	none	unweathered basalt	n.a.	n.a.	n.a.	149 ± 1.8^b^	1101 ± 95^b^
AM	gymnosperm	*S. sempervirens*	7.4 ± 0.1^bc^	non detected^a^	40 ± 20^a^	125 ± 14^ab^	1070 ± 171^b^
AM	gymnosperm	*M. glyptostroboides*	7.7 ± 0.1^c^	10 ± 3.7^ab^	97 ± 39^ab^	109 ± 16^ab^	822 ± 72^ab^
EM	gymnosperm	*P. sylvestris*	6.9 ± 0.2^b^	21 ± 1.9^b^	173 ± 30^bc^	88 ± 8.9^a^	926 ± 236^ab^
EM	angiosperm	*B. pendula*	6.2 ± 0.1^a^	22 ± 1.6^b^	229 ± 9.1^c^	86 ± 9.6^a^	520 ± 31^a^
EM	angiosperm	*N. dombeyi*	7.0 ± 0.1^b^	15 ± 3.5^b^	136 ± 28^b^	87 ± 9.2^a^	868 ± 85^ab^
		one-way ANOVA	*F*_4,30_ = 20.3	*F*_4,24_ = 8.28	*F*_4,24_ = 9.05	*F*_5,26_ = 4.04	*F*_5,26_ = 2.64
			*p* < 0.0001	*p* < 0.0001	*p* < 0.0001	*p* = 0.008	*p* < 0.05

Values sharing the same letter are not statistically different (*p* < 0.05; one-way ANOVA with Tukey multiple comparisons).

^d^Basalt bag pH after 14 months of burial (*n* = 7 ± s.e.m.).

Our experiments with established trees support the hypothesis that both AM and EM fungi direct carbon from plant photosynthesis at silicate rocks containing the highest concentrations of weatherable nutrient elements, and physically degrade mineral surfaces to drive intensification of chemical weathering. This provides the first indication of ‘biosensing’ behaviour by AM fungi, and new field evidence supporting earlier suggestions that EM fungi exhibit similar behaviour based largely on controlled-environment laboratory experiments [12,15]. Across our tree–mycorrhiza types, increased hyphal colonization translates into enhanced basalt weathering (figure 1*b* and table 2). The observation that AM fungi actively weather minerals challenges the recent assumption that they are passive components of the Earth's biological weathering engine [16]. Moreover, it extends the likely importance of mycorrhiza-driven weathering back to the origins of early forests more than 350 Ma, or even to the rise of the first AM-forming land plants more than 50 Myr earlier than this. Our findings provide empirical support for the hypothesis that the Earth's biological weathering engine may have intensified over evolutionary time, while being regulated by the effects of variations in atmospheric CO_2_ and climate. Both control forest productivity, nutrient demand and delivery of photosynthate to roots and mycorrhizas that drive global biotic weathering [17].
